# Emergency Medicine Virtual Conference Participants’ Engagement with Competing Activities

**DOI:** 10.5811/westjem.2021.11.54001

**Published:** 2022-01-03

**Authors:** Deena Khamees, Charles William Kropf, Sarah Tomlinson, James A. Cranford, Michele Carney, Carrie Harvey, Meg Wolff, Mary R.C. Haas, Laura R. Hopson

**Affiliations:** * University of Texas, Department of Emergency Medicine, Houston, Texas; † University of Michigan, Department of Emergency Medicine, Ann Arbor, Michigan; ‡ University of Michigan, Department of Emergency Medicine and Pediatrics, Ann Arbor, Michigan

## Abstract

**Introduction:**

Residency didactic conferences transitioned to a virtual format during the COVID-19 pandemic. This format creates questions about effective educational practices, which depend on learner engagement. In this study we sought to characterize the competitive demands for learner attention during virtual didactics and to pilot methodology for future studies.

**Methods:**

This was a prospective, observational, cohort study of attendees at virtual didactics from a single emergency medicine residency, which employed a self-report strategy informed by validated classroom assessments of student engagement. We deployed an online, two-question survey polling across six conference days using random signaled sampling. Participants reported all activities during the preceding five minutes.

**Results:**

There were 1303 responses over 40 survey deployments across six nonadjacent days. Respondents were residents (63.4%); faculty (27.5%); fellows (2.3%); students (2%); and others (4.8%). Across all responses, about 85% indicated engagement in the virtual conference within the last five minutes of the polls. The average number of activities engaged in was 2.0 (standard deviation = 1.1). Additional activities included education-related (34.2%), work-related (21.1%), social (18.8%), personal (14.6%), self-care (13.4%), and entertainment (4.4%).

**Conclusion:**

Learners engage in a variety of activities during virtual didactics. Engagement appears to fluctuate temporally, which may inform teaching strategies. This information may also provide unique instructor feedback. This pilot study demonstrates methodology for future studies of conference engagement and learning outcomes.

## INTRODUCTION

Emergency medicine (EM) residency programs executed a rapid shift to virtual didactic conferences in response to the safety restrictions resulting from the COVID-19 pandemic. This transition creates questions about effective education, which depends on learner engagement for success.^1^–^3^ Engagement serves as an important and measurable link between instruction and educational outcomes and is defined as focused attention on a specific task.^4^,^5^ Accordingly, multiple direct-observation tools to assess learner engagement and behaviors exist for the classroom.^5^–^7^

Audience engagement with virtual didactics is not well-characterized in the graduate medical education (GME) environment. Related work has focused on asynchronous content and finds short-duration interaction with the resources.^8^–^10^ Information about synchronously delivered virtual content spanning a longer time period is not available. Drawing from existing evidence that the learning environment is a major mediator of learner engagement, it was hypothesized that learners engage in multiple activities during virtual conferences.^4^ In this observational cohort study we sought to characterize the competition for learner attention during virtual didactics and to pilot methodology for assessing engagement in this environment.

## METHODS

### Study Setting and Population

This study occurred at a single, four-year EM residency. Didactics occur weekly in a four-hour block, and content is aimed at residents. Potential participants included all conference attendees: EM residents (N = 64); EM faculty; fellows; medical students; and other guests. The number and composition of attendees fluctuates and on average consists of two-third trainees, one-third faculty, and a small number of others. Sessions were all delivered using the Zoom platform (Zoom Video Communications, San Jose, CA).

### Study Design

This was a prospective, observational cohort study during six weekly didactic blocks between May–August 2020 using a brief survey instrument.

### Survey Instrument

Drawing on self-report methodology for measuring attention and engagement, a brief survey was designed (Supplemental figure 1) through iterative discussion among the authors who have expertise in didactic instruction and survey design.^11^–^13^ We brainstormed possible activities that could be done during conference based on personal experience and feedback from trainees. This initial list was aggregated into broad categories. To enhance construct validity, the resulting list of options was piloted with the pediatric EM fellows during their fellowship didactics and resulted in minor revisions. Data from pilot testing was not included in this study. The final two-question survey was deployed using the Zoom polling feature. Participants identified their role (eg, resident, faculty) and reported all activities performed during the preceding five minutes. The institutional review board granted the study exempt status.

### Study Protocol

Deployment was modeled after a modified, signal-contingent experience sampling method.^14^ The poll was deployed during virtual residency conference. All potential attendees were notified and explicitly informed that responses were anonymous and without consequences. This was done by e-mail prior to the inception of the study and again prior to each day of data collection.

During each of the six conference days, the poll was deployed 4–10 times and was available for 60–75 seconds. Deployment dates were a convenience sample as determined by the conference schedule between May 20–August 5, 2020. Days with extensive small-group breakout sessions or invited external speakers were intentionally avoided. We collected data on the number of participants and time of deployment. Timing varied considerably with deployments during natural breaks in the schedule to minimize disruption of learning. For analysis, polling instances were aggregated into 30-minute blocks from 10 am–2 pm. During the initial three days of polling, 10 polls were distributed each day. Residents provided feedback that this number of polls was intrusive, and on subsequent days the number of polls was reduced to minimize interruption to educational content. As a result of longer sessions in the afternoon (eg, grand rounds and morbidity & mortality), more polls inevitably deployed during the first half of conference to avoid interrupting these longer, more sensitive sessions.

### Data Analysis

We performed descriptive analysis with a focus on trajectories of competing activities (ie, attention) over time. Responses were not linked to individual participants. Analysis was completed with SPSS Statistics software version 27.0 (IBM Corporation, Armonk, NY).

## RESULTS

**Figure 1:**
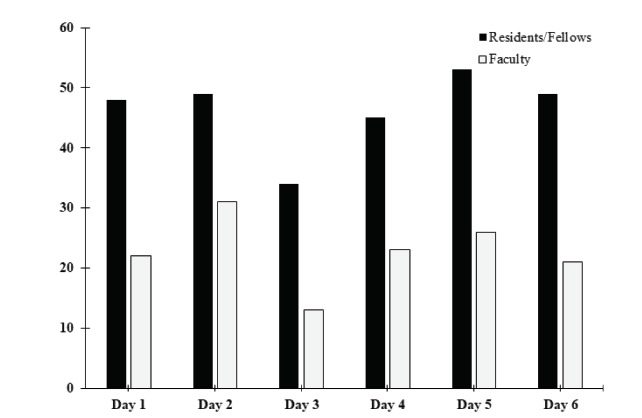
Number of residents/fellows and faculty participants for all study days for assessment of participants’ engagement with competing activities during virtual conference.

There were a total of 1303 responses for 40 polls over six non-consecutive conference sessions encompassing 24 hours of delivered content. Average attendance of residents and faculty over these conferences was 69 participants (46 trainees and 23 faculty) and a small number of others by a self-report process. This data may not accurately reflect the attendance at any given moment or the number available to participate in the poll. Assuming consistent presence in conference, we estimated a response rate of 47% (1303 poll responses/2760 potential respondents averaged over all polls). Figure 1 provides a breakdown of resident and faculty presence during each polling day. Respondents identified as “resident” (63.4%), followed by “faculty” (27.5%), “other” (4.8%), “fellow” (2.3%), and “student” (2.0%). Most polls (75.1%) were conducted in the first half of the conference as noted in the Methods section.

**Figure 2:**
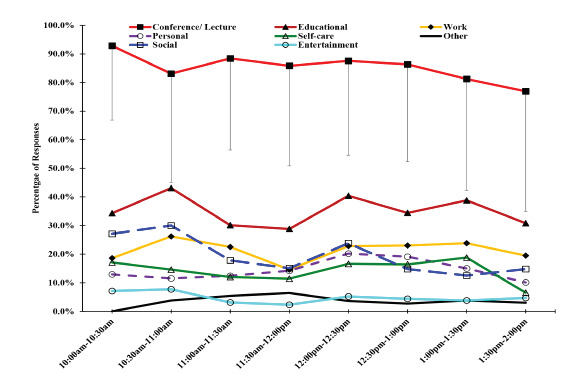
Activities engaged in during the last five minutes by emergency medicine conference attendees, across all polls and all days. Data presented as % of respondents by time of day. Mean conference participation was 85.3% (SD = 35.4%). *EM*, emergency medicine; *SD*, standard deviation.

In total, 69.2% (902/1303) of respondents reported engaging in multiple activities that included the following: education-related (34.2%); work-related (21.1%); social (18.8%); personal (14.6%); self-care (13.4%); entertainment (4.4%); other (4.1%); and driving (0.2%). These categories are defined in the supplemental materials and summarized in Supplemental Table 2. The average number of activities reported on each poll was 2.0 (standard deviation (SD) = 1.1, range 0–8). The relative frequencies of activities by time of day are presented in Figure 2. Participation in polling and reported participation in nearly all activities declined in the second half of conference, except for “work.” The relative distribution of activities also remained stable until the last hour of conference where there appeared to be a downward trend. Given the preliminary nature of this study it was not possible to determine the significance. Of all categories, engagement in educational and social activities varied the most.

## DISCUSSION

Didactic lecture is an essential element of EM education. Our data illustrates that engagement in conference/lecture is consistently high, although it may decline slightly throughout the four-hour session. In addition, learner attention is frequently divided among competing tasks during virtual conference. Literature suggests that multitasking may only be effectively accomplished when the involved behaviors are entirely automatic.^15^ Since didactics are intended to introduce unfamiliar material, competing activities may result in “disruption in the primary task” of conference, which is learning.^15^

One potential solution is the thoughtful incorporation of otherwise competing tasks into didactics, which may decrease task-switching and increase engagement. In a comprehensive review of social media in the classroom, Van Den Beemt et al describe methods to link social media use to intended learning outcomes.^16^ Such an intentional inclusion of social media or any other competing activity may allow participants to bypass the pitfall of unstructured multitasking. Of course, this may be more challenging with personal, high-priority tasks such as childcare. In these circumstances, an additional structured task as described above could represent an added barrier to engagement. Understanding the magnitude and impact on learning of such personal demands may also be an early step in developing solutions.

Educators may be able to use engagement data to more effectively structure conferences to optimize learning. An apparent decline in engagement in the final hour of each conference day was noted. While this finding is of unclear significance within our pilot dataset, if this finding is sustained in more comprehensive work, educators may intentionally plan topics and intervention strategies to increase and sustain engagement such as those involving more active learning approaches during predictable periods of decreased engagement metrics. Examples of applicable techniques include case studies, team-based learning, collaborative learning approaches, and specific tasks to demonstrate higher level learning outcomes.^17^,^18^ Annansingh’s work also suggests that instructional design focused on active learning is particularly important to outcomes in the virtual environment.^18^ Polling techniques, similar to those used in our methodology, and the use of Q&A and chatroom functionalities, may also have utility in engaging attendees in the virtual environment.^19^

Finally, this study demonstrates a pilot methodology for future studies of conference engagement and learning outcomes in the GME environment. Self-reported data on audience activities during a given lecture may serve as useful feedback for programs and presenters. Future work can focus on context-related correlations with engagement as well as exploring implications of this methodology on learning outcomes. For example, an increase in “work” or “entertainment” may indicate disengagement, prompting a deeper probe into the cause.

## LIMITATIONS

There are several potential confounders to our study. The nature of polls appearing abruptly on screen may have artificially increased rates of participation by alerting learners back to the Zoom platform. The self-report nature may have impacted results by minimizing reporting of non-lecture activities (ie, social desirability bias). Additionally, a significant percentage of participants did not respond to polls; absence of response may have been unintentional due to distractions or intentional due to a desire not to participate. It is not possible to calculate what impact this had on our results. Some activities, such as driving, inherently prohibit response, and may be under-represented. It is not possible to account for those who did not answer. There was no technical disruption during conferences at the time of poll deployment, and individual internet connection problems cannot be assessed feasibly. Finally, the content type (eg, lecture, morbidity & mortality, interactive question session) was not controlled for in the analysis. In this pilot study, there was not the capability for this depth of analysis. Learner engagement across the spectrum of virtual lecture types is an area for future research.

## CONCLUSION

Non-conference activities compete for learner attention during virtual residency didactics. This methodology and data could be applied to strategically design conference schedules and the timing of instructional techniques. Our assessment method may also be used to inform feedback to both presenters and programs. Next steps include complementary studies in the in-person didactic setting, multisite reproduction of this study, experimentation with variables such as attendee camera use or educational modality, and an assessment of the correlation between multitasking or task-switching during didactics and learner outcomes.

## Supplementary Information
