# Supplementary material for: Emergency Medicine Virtual Conference Participants’ Engagement with Competing Activities
Source: West J Emerg Med. 2022 Jan 3;23(1):103–7. doi: 10.5811/westjem.2021.11.54001 (PMC8782122; doi:10.5811/westjem.2021.11.54001)
Supplement: Supplementary file 1 [file wjem-23-103-s001.docx]

**Appendices**

**Supplemental Figure 1. Survey**

In the last 5 minutes, in which of the following activities have you engaged? Select all that apply.

1. Conference lecture
2. Educational (Zoom/conference chat, web browsing related to conference content),
3. Work (work-related tasks/projects, chart completion, work email)
4. Social (texting/personal messaging, social media, personal email)
5. Entertainment (web surfing, TV/movie/streaming, books/magazines, games/puzzles, arts/crafts)
6. Driving
7. Personal (e.g. interacting with family, childcare, pet care, household chores)
8. Self care (e.g. eating, showering, sleeping, exercise)
9. Other”

How do you describe yourself?

1. Student
2. Resident
3. Fellow
4. Faculty
5. Other

**Supplemental Table 2. Frequency of Activities Engaged in Over the Last 5 Minutes By All Participants.**

| ***“In the last 5 minutes, in which of the following activities have you engaged?”*** | ***N*^1^ (%)** |
| --- | --- |
| **1. Conference/Lecture** | **1,112 (85.3%)** |
| **2. Educational** | **445 (34.2%)** |
| **3. Work** | **275 (21.1%)** |
| **4. Social** | **245 (18.8%)** |
| **5. Personal** | **190 (14.6%)** |
| **6. Self-Care** | **175 (13.4%)** |
| **7. Entertainment** | **57 (4.4%)** |
| **8. Other** | **55 (4.2%)** |

**^1^Based on a total of 1,303 responses and 2,554 reported activities.**
